# Assessing biomimetic aquaporin membrane for forward osmosis desalination process: A dataset

**DOI:** 10.1016/j.dib.2019.104482

**Published:** 2019-09-04

**Authors:** Soleyman Sahebi, Nasim Fadaie, Leila Mirshekar, Bahram Kamarehie, Toraj Mohammadi

**Affiliations:** a Department for Management of Science and Technology Development, Ton Duc Thang University, Ho Chi Minh City, Viet Nam; b Faculty of Environment and Labour Safety, Ton Duc Thang University, Ho Chi Minh City, Viet Nam; c Research and Technology Centre of Membrane Processes (RTCMP), School of Chemical, Petroleum and Gas Engineering, Iran University of Science and Technology (IUST), Narmak, Tehran, Iran; d Nutrition Health Research Centre and Department of Environmental Health, School of Health and Nutrition, Lorestan University of Medical Sciences, Khorramabad, Iran

**Keywords:** Forward osmosis, Aquaporin, Biomimetic membrane, Desalination, Fertigation

## Abstract

This paper presents the performance of aquaporin forward osmosis membrane using chemical fertilizers as a draw solution. The comprehensive evaluation conducted for five conventional fertilizers ((CO (NH_2_)_2_, KCl, CaCl_2_, (NH_4_)_2_SO_4_) and (NH_4_)_2_HPO_4_) as draw solutions. The diluted fertilizer can be used directly for farming as fertigation. In this process, DSs do not need to be recovered and it is a single step desalination process. The data include the characterization of the intrinsic properties of the membrane samples and their performance under FO and PRO modes of operations. In addition, the data for various draw solution concentration under feed solution with deferent total dissolved solids (TDS) were evaluated. For example, a water flux of 17.5 L m^− 2^ h^− 1^ and 23.92 g m^−2^ h^−1^ reverse solute flux (RSF) was achieved under the FO operation mode for 3 M KCl.

**Table:** Specifications Table

Subject area	Separation and purification technology
More specific subject area	Forward osmosis, Biomimetic thin film composite membrane
Type of data	Figure, table, schematic
How data was acquired	Schottky Field Emission Scanning Electron Microscope (SEM, Zeiss Supra 55VP, Carl Zies AG, Germany Optical Tensiometer (Attension Theta Lite 100, Biolin Scientific, Finland)
Data format	Raw and Analyzed
Experimental factors	Performance new Aquaporin forward osmosis membrane was evaluated with five fertilizers as draw solution and feed solution with different TDS.
Experimental features	The desalinated water containing diluted fertilizer to be used directly or after further dilution for farm fertigation.
Data source location	Research and Technology Centre of Membrane Processes (RTCMP), School of Chemical, Petroleum and Gas Engineering, Iran University of Science and Technology (IUST), Narmak, Tehran, Iran Department of Environmental Health, School of Health and Nutrition, Lorestan University of Medical Sciences, Khorramabad, Iran Department for Management of Science and Technology Development, Ton Duc Thang University, Ho Chi Minh City, Vietnam Faculty of Environment and Labour Safety, Ton Duc Thang University, Ho Chi Minh City, Vietnam
Data accessibility	The raw data files are provided in the supplementary file. All other data is within this article.

**Table:** 

**Value of the data** • FO process requires much lower energy than the existing desalination technologies. • Energy requirement is even lower when the diluted draw solution (fertilizers) can be used directly for farm irrigation. • This data provides valuable information and gives more insights on the newly commercialized Aquaporin FO membrane. • The key parameters in utilizing fertilizer as a draw solution applying biomimetic FO membrane were explored.

## 1 Data

**Fig. 1:**
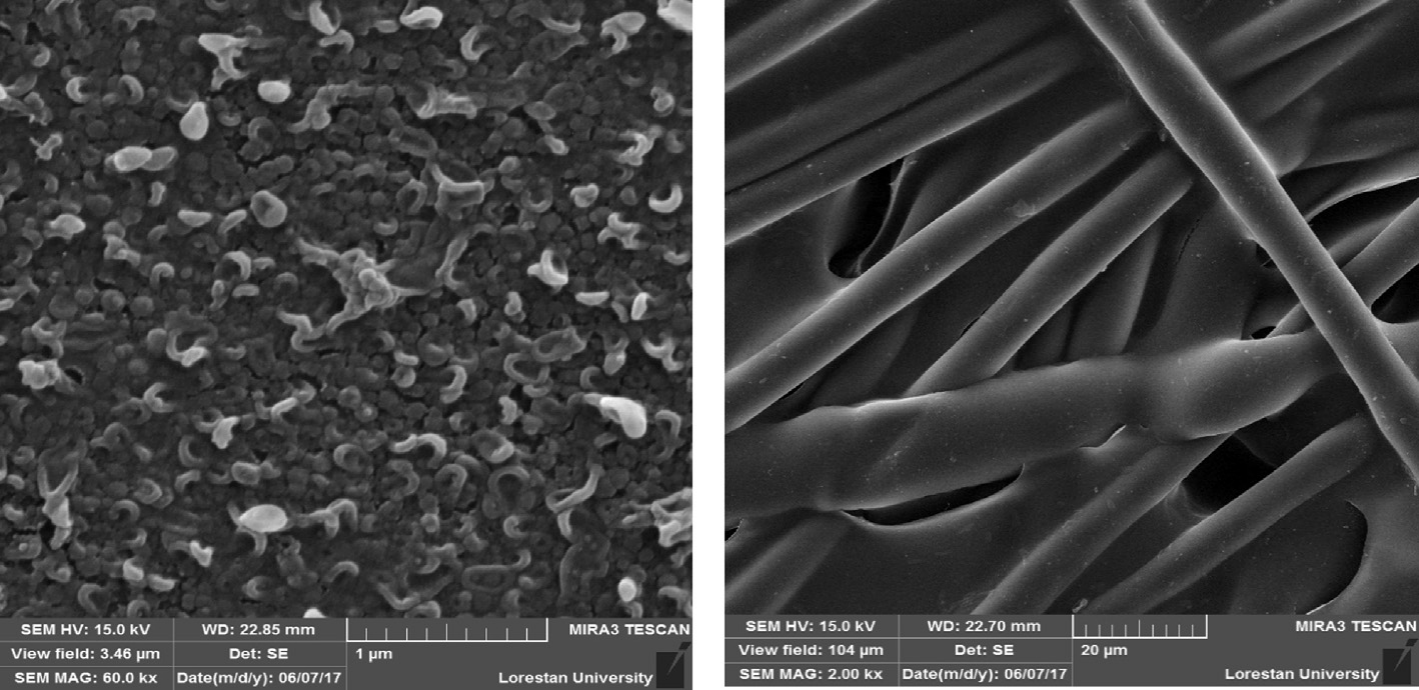
SEM images for the top rejection layer and non-woven backing fabric support for the virgin Aquaporin FO membrane in this study.

Fig. 1 presents the SEM images for the top surface rejection layer and non-woven backing fabric support for Aquaporin FO membrane. The Biomimetic FO membrane top surface (rejection layer) which is formed by AQPs proteins is similar to conventional thin film composite membrane polyamide layer in terms of topology.

**Fig. 2:**
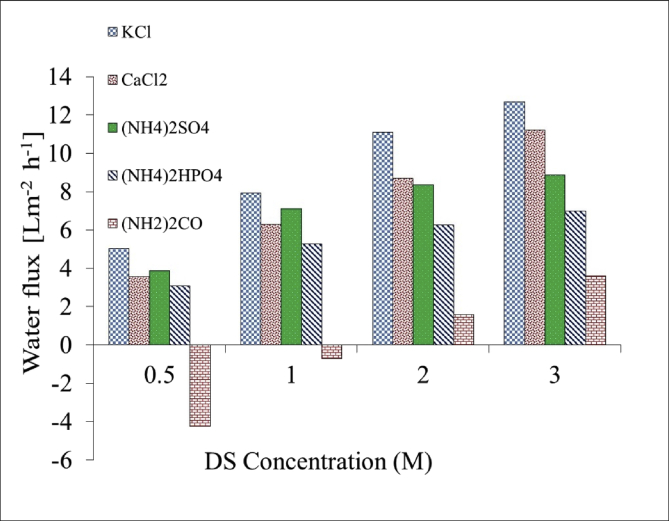
Comparison of the water flux for different fertilizers as a DS at different concentrations using 10000 mg/L NaCl as feed solution in the FO mode.

**Fig. 3:**
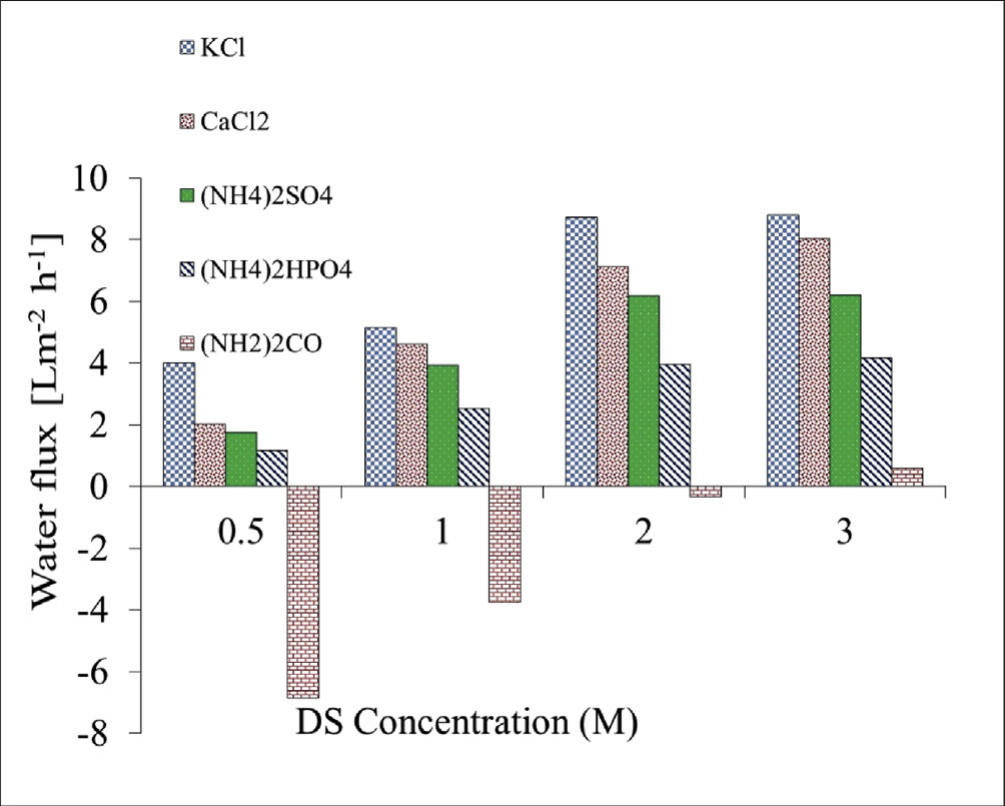
Comparison of the water flux for different fertilizers as a DS at different concentrations using 20000 mg/L NaCl as feed solution in FO mode.

**Fig. 4:**
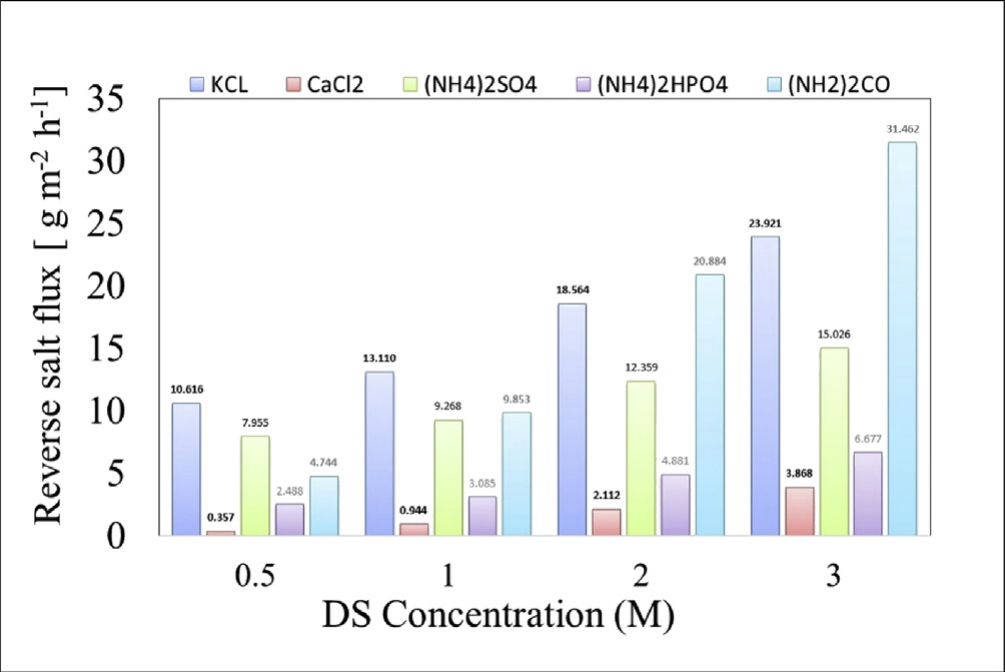
Comparison of RSF of five fertilizers as DS at different concentrations using DI water as FS in the FO mode.

**Fig. 5:**
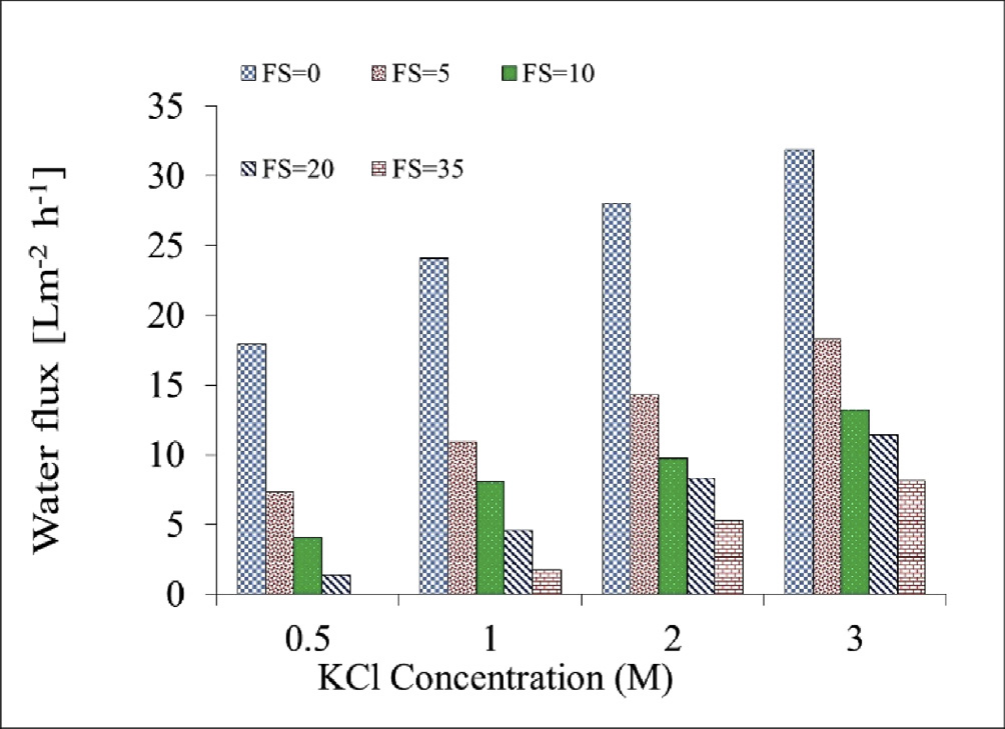
Performance of KCl in terms of water flux at different M concentrations of DS and FS with 0–5–10–20–35 g/L NaCl.

**Fig. 6:**
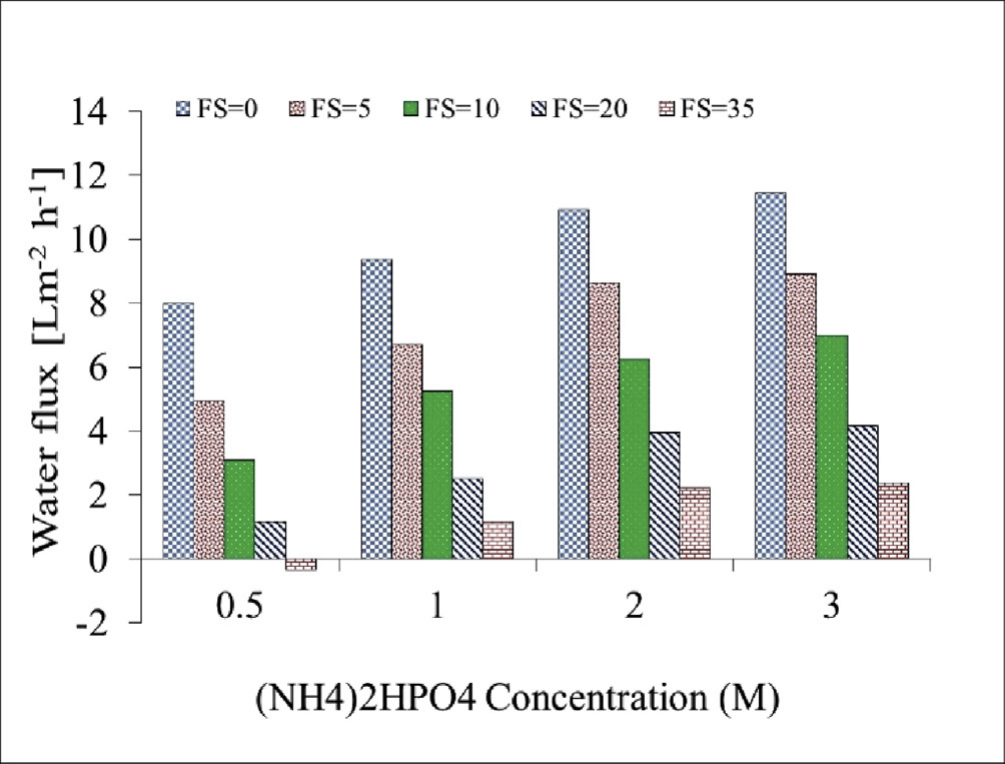
Performance of (NH4)2HPO4 in terms of water flux at different M concentrations of DS and FS with 0–5–10–20–35 g/L NaCl.

**Fig. 7:**
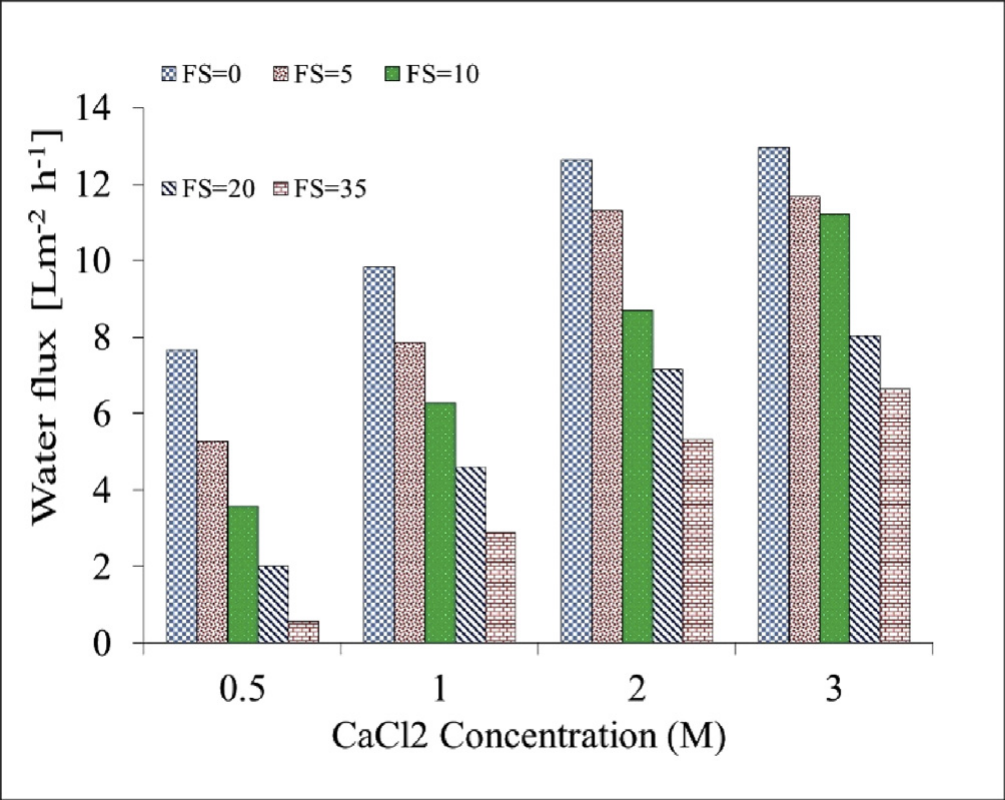
Performance of CaCl2 in terms of water flux at different M concentrations of DS and FS with 0–5–10–20–35 g/L NaCl.

**Fig. 8:**
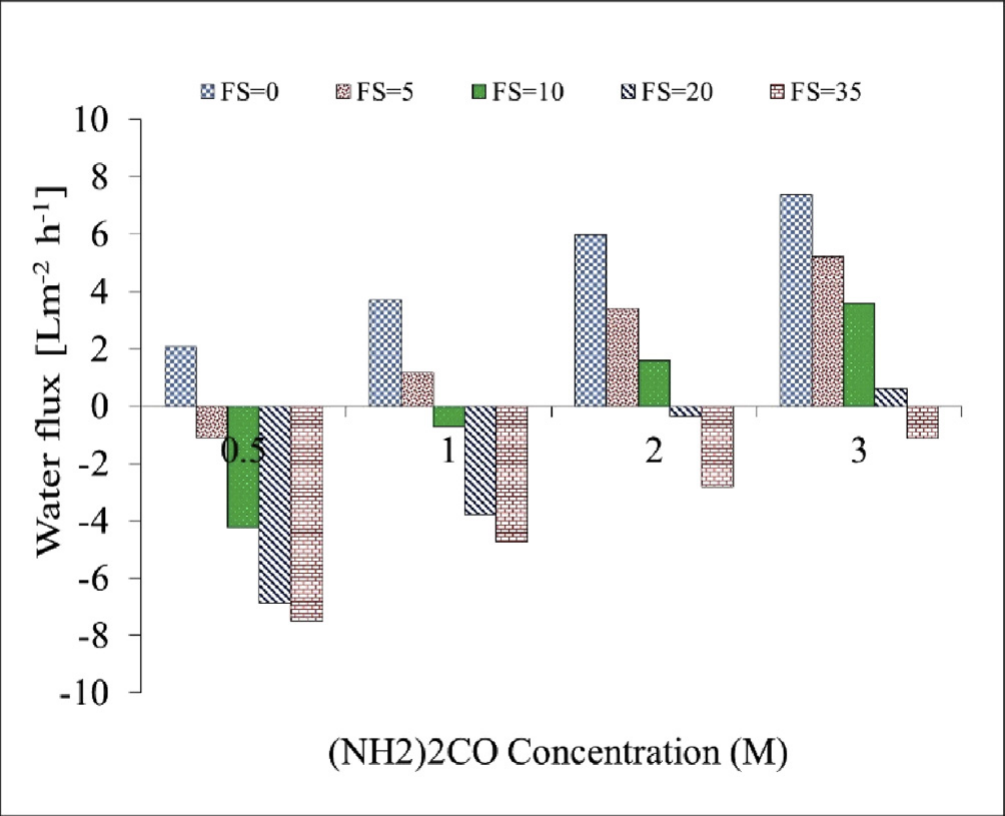
Performance of Urea in terms of water flux at different M concentrations of DS and FS with 0–5–10–20–35 g/L NaCl.

**Fig. 9:**
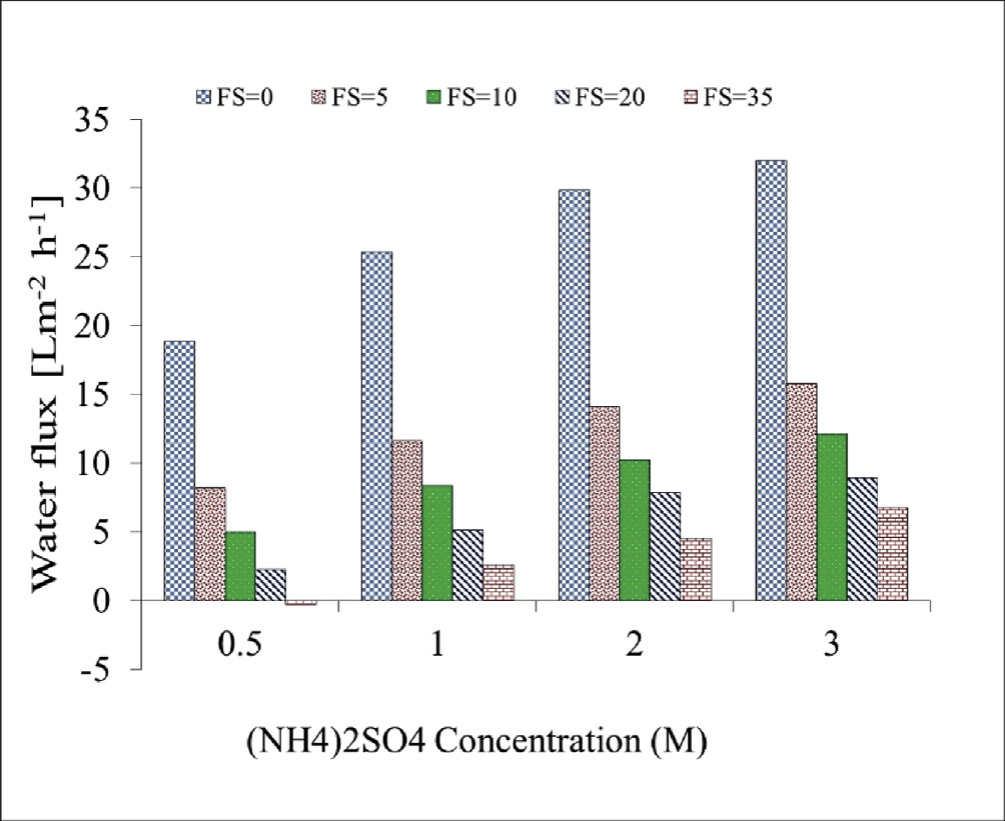
Performance of (NH4)_2_SO_4_ in terms of water flux at different Molar concentrations of DS and FS with 0–5–10–20–35 g/L NaCl.

Table 1. Presents the AQPs membrane characterization. Fig. 2, Fig. 3 present the membrane performance in terms of water flux in the FO mode with five different fertilizers as DS different using 10 gr/L and 20 gr/L NaCl as FS, respectively. The performance was in the following order: KCl>(NH_4_)2SO_4_>CaCl_2_>(NH_4_)2HPO_4_>Urea. Fig. 4 also shows the amount of wasting draw solute through phenomenon called reverse solute flux (RSF). The RSF plays a central role in the valuation of the FO process in terms of economic measure and membrane fouling tendency. Fig. 5, Fig. 6, Fig. 7, Fig. 8, Fig. 9 present the performance of each specific DS with five different concentration of feed solution (0–5–10–20–35 g/L NaCl) in terms of water flux in FO operation mode.

**Table 1:** Characterisation of AQPs FO membrane.

Membrane ID	Thickness (μm)	Porosity (%)	Contact angle (º)
			Active layer
AQPs	135 ± 2.0	69	51

**Fig. 10:**
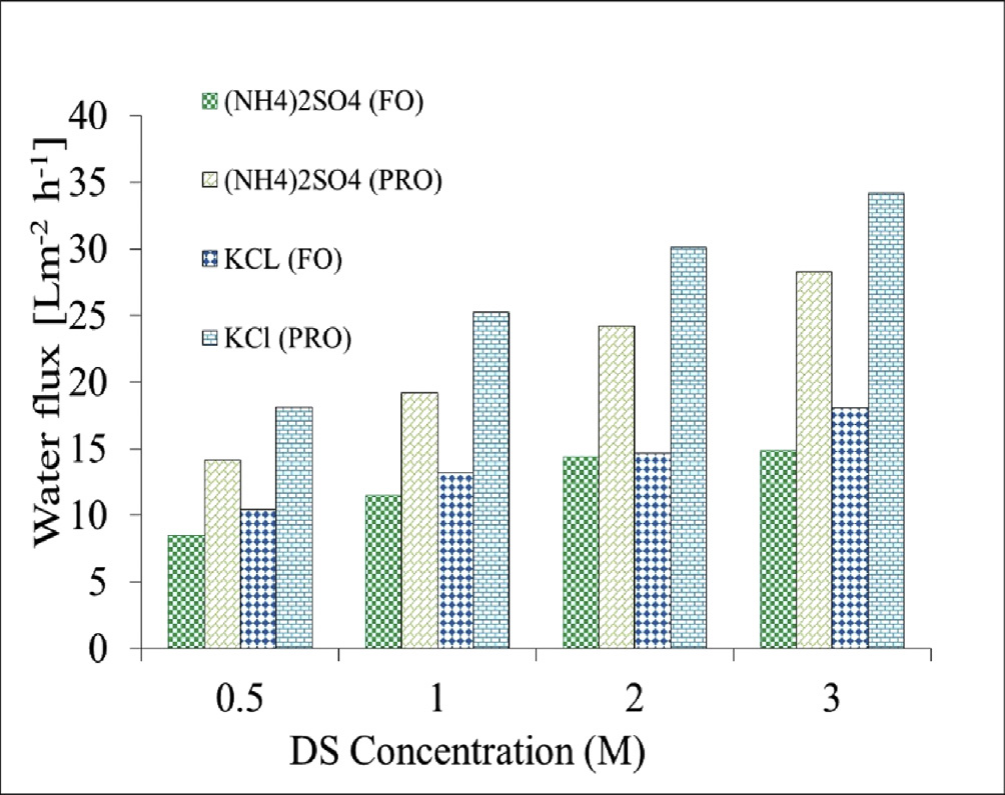
Comparing water flux in FO and PRO modes using DI water as FS at different Molar concentration for KCl and (NH4)_2_SO_4_.

Finally, Fig. 10 shows the membrane performance in FO and PRO modes using DI water as FS at different Molar concentration for 2 selected fertilizers, KCl and (NH4)_2_SO_4_. In the PRO mode, the net gain water flux was more compared to the FO mode. This indicates the effect of ICP in the PRO mode is less than FO mode of operation. The raw data presented in the supplementary file as well.

## 2 Experimental design, materials, and methods

### 2.1 Feed and draw solutions preparation

(NH_2_)_2_CO, KCl, CaCl_2_, (NH_4_)_2_SO_4_, (NH_4_)_2_HPO_4_ were used as draw solutions provided by (SAMCHUN, Korea). 0.5, 1, 2, and 3 M molar concentration levels were prepared. DI water and NaCl (5-10-20-35 g/L) were prepared as a feed solution (FS).

### 2.2 Measurement of intrinsic properties of the membrane

Intrinsic properties of the Biomimetic-FO membrane were determined by RO testing mode by utilizing the FO membrane cell and applying hydraulic pressure (0–5 bar). Water permeability (*A* value) was calculated based on the following equation:

(1) $$A=\frac{\text{Δ}V_{a}}{\text{Δ}t_{a}\times A_{m}\times \text{Δ}P}$$

Water permeability was obtained by placing DI water in the FS container and adjusting hydraulic pressure of 5.0 bar. Δ_*Va*_ is the acquired permeate water over a specific time, Δ*P* is the applied hydraulic pressure difference and *Am* and Δ*ta* is the membrane area.

NaCl rejection property of the Biomimetic-FO membrane was calculated by the following equation.

(2) $$R=\frac{Cf-Cp}{Cf}\times 100\%$$

where *C*_*f*_ and *C*_*p*_ are the amount of NaCl concentrations for the feed and permeate container [1], [2].

The salt permeability coefficient (B) was calculated by the following equation:

(3) $$B=\frac{A\left(1-R\right)\left(\text{Δp}-\text{Δ}\pi \right)}{R}$$

where A is water permeability, *R* is the salt rejection, Δ*p* is the applied pressure and Δπ is the osmotic pressure difference for the Aquaporin FO membrane [3], [4], [5], [6].

### 2.3 Substrate characterization

The membrane morphologies were assessed using (FE-SEM, MIRA3-LMU model, Czech Republic) a high-resolution Schottky Field Emission Scanning Electron Microscope.

Membrane porosity (ε) was calculated by weighing the wet mass (W_1_) and the dry mass (W_2_) of membrane samples based on the following equation:

(4) $$\varepsilon =\frac{\left(W1-W2\right)/\rho i}{\left[\frac{W1-W2}{\rho i}\right],+\left[W2/\rho m\right]}\times 100\%$$

where ρi is the density of the used wetting solvent, and ρm is the density of the membrane sample. The thickness of the membranes was measured by a digital micrometer (293-330 Mitutoyo, Japan).

### 2.4 Membrane tests under FO and PRO processes

**Fig. 11:**
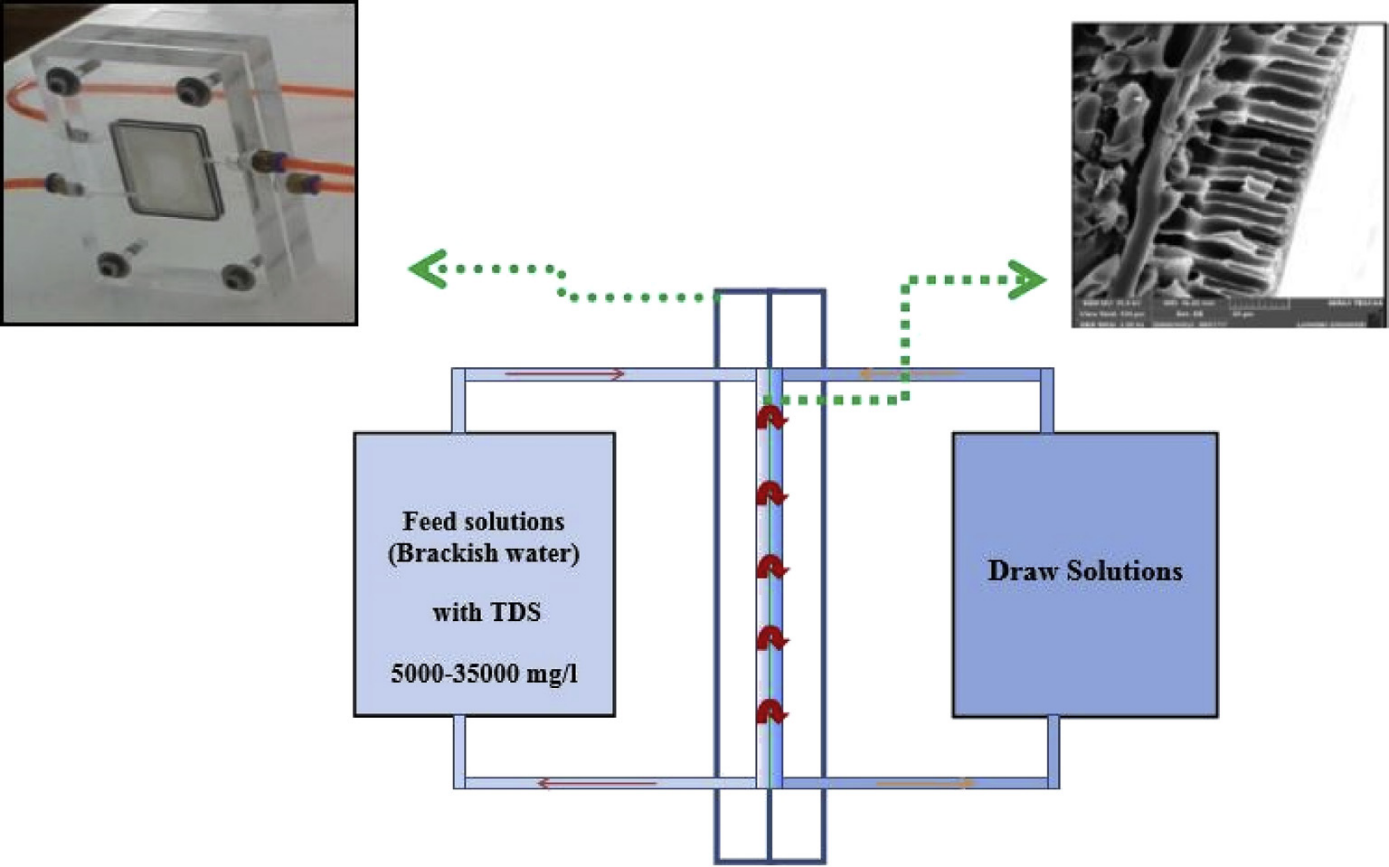
Schematic of the lab-scale FO experimental setup.

**Fig. 12:**
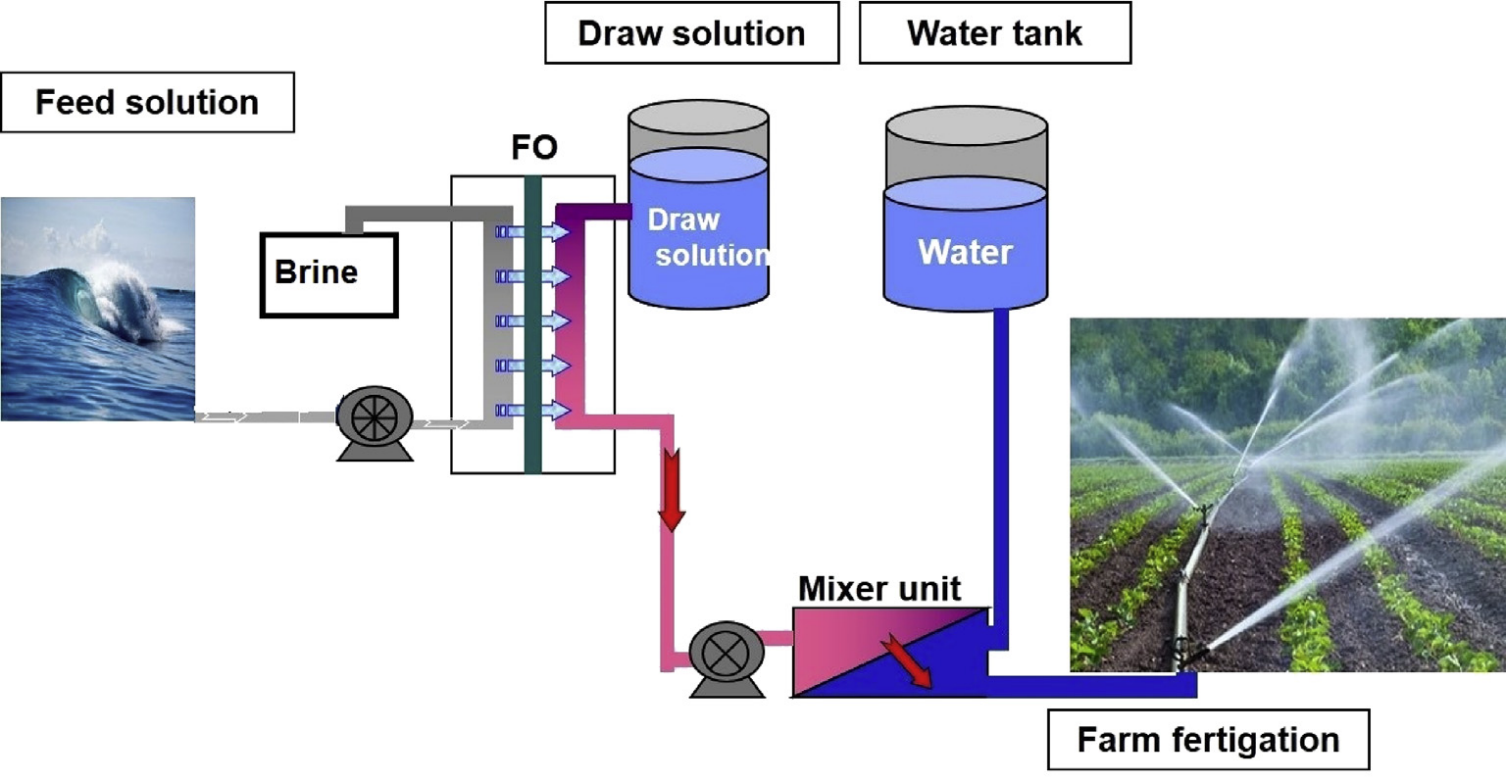
Schematic of using brackish water for farm fertigation via FO process.

Performance tests under the FO and PRO processes were assessed in the designed FO cell presented in Fig. 11 provides a useful area of 6.2 cm^2^ with 2 cm in width, 3.1 cm in length and 0.3 cm in depth on both sides. The reverse solute flux (RSF) was assessed by observing the electrical conductivity (EC) applying a multimeter (Lutron-CD4303, Germany) where deionized water is used as feed water [7], [8] (see Fig. 12).

## 3 Application for fertigation

The desalinated water via FO process using fertilizer as a draw solution can be used directly for farming as fertigation [9], [10]. In this process, draw solutions do not need recovery and directly will be used for farming [11], [12]. Thus that is a single-stand desalination process [10], [11].
