# Supplementary material for: Assessing biomimetic aquaporin membrane for forward osmosis desalination process: A dataset
Source: Data Brief. 2019 Sep 4;26:104482. doi: 10.1016/j.dib.2019.104482 (PMC6811868; doi:10.1016/j.dib.2019.104482)
Supplement: Multimedia component 1 [file mmc1.docx]

**Supplementary file: assessing biomimetic aquaporin membrane for forward osmosis desalination process:** [**A dataset**](https://www.sciencedirect.com/science/article/pii/S2352340918312241)

Soleyman Sahebi^1,2*^, Nasim Fadaie^3^, Mirshekar J.L^4^, B. Kamarehi ^4^*, T. Mohammadi^3^*

*^1^Department for Management of Science and Technology Development, Ton Duc Thang University, Ho Chi Minh City, Vietnam ^2^Faculty of Environment and Labour Safety, Ton Duc Thang University, Ho Chi Minh City, Vietnam*

*^3^Research and Technology Centre of Membrane Processes (RTCMP), School of Chemical, Petroleum and Gas Engineering, Iran University of Science and Technology (IUST), Narmak, Tehran, Iran*

*4Nutrition Health Research Centre and Department of Environmental Health, School of Health and Nutrition, Lorestan University of Medical Sciences, Khorramabad, Iran*

**Corresponding authors:* [torajmohammadi@iust.ac.ir](mailto:torajmohammadi@iust.ac.ir)*;* [*soleyman.sahebi@tdtu.edu.vn*](mailto:soleyman.sahebi@tdtu.edu.vn)*;* [*b.kamarehie@Lums.ac.ir*](mailto:b.kamarehie@Lums.ac.ir)

**Raw data are presented as supplementary file.**

**Fig 2.** Comparison of the water flux for different fertilizers as a DS at different concentrations using 10000 mg /L NaCl as feed solution in the FO mode.

| **(NH2)2CO** | **(NH4)2HPO4** | **(NH4)2SO4** | **CaCl2** | **KCl** | **C (M)** |
| --- | --- | --- | --- | --- | --- |
| -4.232 | 3.084 | 3.868 | 3.571 | 5.052 | 0.5 |
| -0.706 | 5.261 | 7.113 | 6.274 | 7.935 | 1 |
| 1.584 | 6.261 | 8.355 | 8.697 | 11.106 | 2 |
| 3.597 | 7 | 8.852 | 11.223 | 12.677 | 3 |

**Fig 3.** Comparison of the water flux for different fertilizers as a DS at different concentrations using 20000 mg /L NaCl as feed solution in FO mode.

| **(NH2)2CO** | **(NH4)2HPO4** | **(NH4)2SO4** | **CaCl2** | **KCl** | **C (M)** |
| --- | --- | --- | --- | --- | --- |
| -6.868 | 1.165 | 1.742 | 2.019 | 4.003 | 0.5 |
| -3.768 | 2.510 | 3.919 | 4.623 | 5.155 | 1 |
| -0.348 | 3.965 | 6.187 | 7.145 | 8.735 | 2 |
| 0.594 | 4.171 | 6.210 | 8.039 | 8.794 | 3 |

**Fig. 4.** Comparison of RSF of five fertilizers as DS at different concentrations using DI water as FS in the FO mode.

| **Fertilizers** | **C (M)** | **0.5** | **1** | **2** | **3** |
| --- | --- | --- | --- | --- | --- |
| **KCl** | | 10.616 | 13.110 | 18.564 | 23.921 |
| **CaCl2** | | 0.357 | 0.944 | 2.112 | 3.868 |
| **(NH4)2SO4** | | 7.955 | 9.268 | 12.359 | 15.026 |
| **(NH4)2HPO4** | | 2.488 | 3.085 | 4.881 | 6.677 |
| **Urea** | | 4.744 | 9.853 | 20.884 | 31.462 |

**Fig. 5.** Performance of KCl in terms of water flux at different M concentrations of DS and FS with 0-5-10-20-35 gram/L NaCl.

| **FS=35** | **FS=20** | **FS=10** | **FS=5** | **FS=0** | **Concentrations (M)** |
| --- | --- | --- | --- | --- | --- |
| -1.048 | 1.365 | 4.068 | 7.310 | 17.926 | 0.5 |
| 1.748 | 4.594 | 8.061 | 10.906 | 24.061 | 1 |
| 5.271 | 8.290 | 9.774 | 14.274 | 28.019 | 2 |
| 8.139 | 11.423 | 13.248 | 18.303 | 31.839 | 3 |

**Fig. 6.** Performance of (NH4)2HPO4 in terms of water flux at different M concentrations of DS and FS with 0-5-10-20-35 gram/L NaCl.

| **FS=35** | **FS=20** | **FS=10** | **FS=5** | **FS=0** | **Concentrations (M)** |
| --- | --- | --- | --- | --- | --- |
| -0.345 | 1.165 | 3.084 | 4.942 | 7.997 | 0.5 |
| 1.177 | 2.510 | 5.261 | 6.713 | 9.374 | 1 |
| 2.210 | 3.965 | 6.261 | 8.619 | 10.916 | 2 |
| 2.387 | 4.171 | 7.000 | 8.923 | 11.448 | 3 |

**Fig. 7.** Performance of CaCl2 in terms of water flux at different M concentrations of DS and FS with 0-5-10-20-35 gram/L NaCl.

| **FS=35** | **FS=20** | **FS=10** | **FS=5** | **FS=0** | **Concentrations (M)** |
| --- | --- | --- | --- | --- | --- |
| 0.558 | 2.019 | 3.571 | 5.281 | 7.674 | 0.5 |
| 2.881 | 4.623 | 6.274 | 7.865 | 9.845 | 1 |
| 5.319 | 7.145 | 8.697 | 11.303 | 12.632 | 2 |
| 6.632 | 8.039 | 11.223 | 11.674 | 12.948 | 3 |

**Fig. 8.** Performance of Urea in terms of water flux at different M concentrations of DS and FS with 0-5-10-20-35 gram/L NaCl.

| **FS=35** | **FS=20** | **FS=10** | **FS=5** | **FS=0** | **Concentrations (M)** |
| --- | --- | --- | --- | --- | --- |
| -7.487 | -6.868 | -4.232 | -1.106 | 2.081 | 0.5 |
| -4.735 | -3.768 | -0.706 | 1.158 | 3.703 | 1 |
| -2.819 | -0.348 | 1.584 | 3.419 | 5.987 | 2 |
| -1.126 | 0.594 | 3.597 | 5.223 | 7.381 | 3 |

**Fig. 9.** Performance of (NH4)_2_SO_4_ in terms of water flux at different Molar concentrations of DS and FS with 0-5-10-20-35 gram/L NaCl.

| **FS=35** | **FS=20** | **FS=10** | **FS=5** | **FS=0** | **Concentrations (M)** |
| --- | --- | --- | --- | --- | --- |
| -0.284 | 2.252 | 4.977 | 8.213 | 18.842 | 0.5 |
| 2.613 | 5.106 | 8.358 | 11.581 | 25.355 | 1 |
| 4.561 | 7.842 | 10.239 | 14.119 | 29.865 | 2 |
| 6.755 | 8.906 | 12.148 | 15.742 | 32.006 | 3 |

**Fig. 10.** Comparing water flux in FO and PRO modes using DI water as FS at different Molar concentration for KCl and (NH4)_2_SO_4_.

| **Concentrations** | **(NH4)2SO4** | | **KCl** | |
| --- | --- | --- | --- | --- |
| (Molar) | FO | PRO | FO | PRO |
| 0.5 | 8.468 | 14.2 | 10.458 | 18.1 |
| 1 | 11.461 | 19.2 | 13.123 | 25.2 |
| 2 | 14.345 | 24.2 | 14.661 | 30.1 |
| 3 | 14.871 | 28.3 | 18.019 | 34.2 |
